# Molecular motor KIF3B in the prelimbic cortex constrains the consolidation of contextual fear memory

**DOI:** 10.1186/s13041-021-00873-9

**Published:** 2021-11-08

**Authors:** Nadine F. Joseph, Aya Zucca, Jenna L. Wingfield, Isabel Espadas, Damon Page, Sathyanarayanan V. Puthanveettil

**Affiliations:** 1 The Skaggs Graduate School of Chemical and Biological Sciences, Scripps Research Institute, La Jolla, CA 92037 USA; 2 Department of Neuroscience, The Scripps Research Institute, Jupiter, FL 33458 USA

**Keywords:** Kinesins, Contextual fear memory, Extinction, Dendritic spines

## Abstract

Molecular and cellular mechanisms underlying the role of the prelimbic cortex in contextual fear memory remain elusive. Here we examined the kinesin family of molecular motor proteins (KIFs) in the prelimbic cortex for their role in mediating contextual fear, a form of associative memory. KIFs function as critical mediators of synaptic transmission and plasticity by their ability to modulate microtubule function and transport of gene products. However, the regulation and function of KIFs in the prelimbic cortex insofar as mediating memory consolidation is not known. We find that within one hour of contextual fear conditioning, the expression of KIF3B is upregulated in the prelimbic but not the infralimbic cortex. Importantly, lentiviral-mediated knockdown of KIF3B in the prelimbic cortex produces deficits in consolidation while reducing freezing behavior during extinction of contextual fear. We also find that the depletion of KIF3B increases spine density within prelimbic neurons. Taken together, these results illuminate a key role for KIF3B in the prelimbic cortex as far as mediating contextual fear memory.

**Supplementary Information:**

The online version contains supplementary material available at 10.1186/s13041-021-00873-9.

## Introduction

Due to the prevalence of, and lack of effective treatments for, fear-based disorders such as posttraumatic stress disorder (PTSD), there is a heightened interest and a clinical relevance found in understanding the neural basis of fear memory regulation. Traditionally, Pavlovian fear conditioning and extinction have been used to study the neural regulation of fear responses [1–3]. Contextual fear conditioning (CFC) is a learning paradigm in which an association is formed between a context and a mild electric foot shock called the unconditioned stimulus (US) [2]. This differs from extinction learning, where repeated presentations of a conditioned stimulus (CS) without the unconditioned stimulus (US) lead to a gradual decrease in the conditioned response (CR) [4, 5]. Extinction is a form of new inhibitory learning that is context-dependent [5].

A large body of research has implicated the medial prefrontal cortex in fear memory [6–16]. The medial prefrontal cortex is divided into two major subregions: the prelimbic (PrL) and infralimbic (IL) cortices. Tracing and lesion studies have found that these subregions appear to have opposing roles in the context of fear memory expression and extinction. For instance, Burgos-Robles et al. found that activity of PrL neurons correlated with freezing behavior occurring during cued fear conditioning [17]. In addition, microstimulation of the PrL resulted in enhanced freezing during fear expression and extinction [18]. Similarly, PrL inactivation blocked the expression of learned fear [19, 20]. These findings greatly differ from those characterizing the IL. Early work from Milad and Quirk, for instance, found that neurons in the IL fire during the CS presentation only after the CS-US association has been extinguished [12]. Likewise, inactivation of the IL with muscimol prevented the acquisition and retention of extinction memory [19]. These studies demonstrate that the PrL appears to control fear expression [9, 20–22] while the IL appears to promote fear extinction [6, 18, 21, 23, 24].

Though there is a great deal of literature regarding the contribution of the PrL to fear memory expression, the underlying molecular mechanisms that support the consolidation of these memories remain poorly understood. We recently showed that encoding of contextual memories induces new protein synthesis, and that inhibition of translation, or Homer3, in the PrL produces deficits in the consolidation of contextual fear [25]. To better understand the molecular underpinnings of the PrL in contextual fear, we searched for known regulators of synapse function and morphogenesis, as it is well known that learning requires structural changes at the synapse [26–28].

It has been broadly demonstrated that KIFs are important for structural plasticity [29–31] as well as memory [31–33]. Our own lab demonstrated that KIF11 KD (knockdown) in hippocampal neurons increased dendritic branching without a change in spine density [29]. Similarly, it has been shown that knockdown of KIF3B in cortical neurons causes an increase in dendritic arborization, with an increase in mushroom and thin spines as well [30]. In contrast, KIF21B KD neurons have reduced dendritic branching [31], which also includes a reduction in mushroom and stubby spines. It is worth noting that KIF21B-null mice display memory deficits [31], and that KIF17 knockout mice also exhibit impairments in several different memory types. These findings comprehensively highlight the interplay between structural plasticity and memory.

In this manuscript, we show that the expression of KIF3B is upregulated in the PrL and that CFC is impaired with KIF3B knockdown. In addition, we found that KIF3B knockdown within the PrL reduces freezing during extinction learning, and we also observed that KIF3B depletion increases spine density in the PrL.

## Results

### Contextual fear conditioning results in an upregulation of the expression of KIF3B mRNAs in prelimbic neurons

To identify KIFs that could play a unique role in contextual fear memory, we selected 12 KIFs based on their expression in the brain as well as their previously known functions. This included those involved in cargo transport (i.e., KIF3A, 3B, 5A, 5B, 5C, 17) [33, 34] and microtubule regulation (i.e., KIF 11, 21A and 21B) [29, 35–37]. Recalling our previous study in the sea slug *Aplysia*, which indicated that KIFs are physiologically upregulated during learning [32], we reasoned that learning and memory may also regulate the expression of specific KIFs within the PrL. To test this hypothesis, we employed qRT-PCR to determine whether the KIFs’ expression would be altered with CFC.

**Fig. 1:**
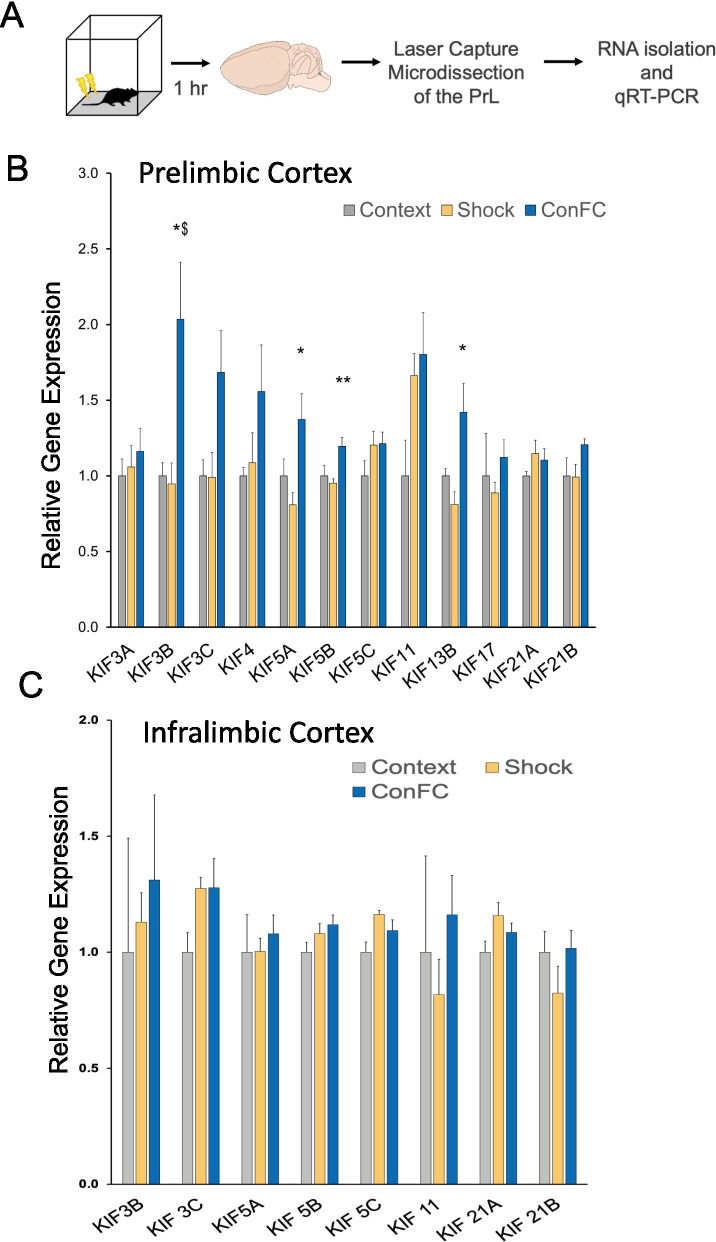
Expression of KIF3B is upregulated within the PrL during the consolidation of contextual fear memory. **A** Experimental schema. **B** Analysis of kinesin expression from RNA derived from tissue of the PrL 1 h after contextual fear conditioning. Ct values were normalized to 18 s RNA for dCT and ddCT was calculated relative to the shock group levels. One-way ANOVA; *p < 0.05 with Tukey post-hoc test, ^$^p < 0.05 vs. context; *p < 0.05 vs. shock; error bars are SEM. N = 3–5 per group. **C** Analysis of kinesin expression of RNA derived from tissue of the IL 1 h after contextual fear conditioning. Data used for preparing plots are shown in Additional file 1: Table S1

To carry out this screen, nine-week-old C57Bl6/J mice underwent CFC. One hour later, the mice were sacrificed, the brains sectioned, and the entire PrL was isolated via laser capture microdissection (LCM) of brain slices. Total RNAs were isolated from LCM sections and analyzed by qRT-PCR (Fig. 1A). We found that, of the kinesins that were tested, KIF3B expression in the prelimbic cortex was significantly upregulated when the CFC group (2.03 ± 0.40) was compared to both shock- (0.95 ± 0.14) and context-only controls (1.00 ± 0.09) (one-way ANOVA, Tukey post-hoc test, F_2,9_ = 6.651 *p < 0.05, Fig. 1B). KIF3B is a mitotic KIF whose functions in post-mitotic neurons, particularly in the PrL, remains poorly understood.

To assess the specificity of the regulation of KIFs by CFC, we next examined the expression of a subset of KIFs in the infralimbic cortex (IL). These KIFs were selected based on their significant upregulation in fear conditioned animals compared to shock alone and context alone controls. Interestingly, we did not find an upregulation of these KIFs in the RNAs isolated from LCM sections containing IL (CFC group 1.205 ± 0.10 and shock-only 1.103 ± 0.03 and context-only 1.00 ± 0.11, one-way ANOVA, F_2,11_ = 1.348 p > 0.05, Fig. 1C). Taken together, these results indicate KIF3B is selectively upregulated in the prelimbic cortex following CFC.

### Knockdown of KIF3B within the PrL has no effect on general locomotion or anxiety-like behavior

The upregulation of KIF3B suggested that KIF3B expression might be critical for contextual fear memory. Therefore, we assumed that depletion of KIF3B could inhibit consolidation of contextual fear. To deplete KIF3B within the PrL, we prepared lentiviral particles expressing KIF3B shRNA (LV-shKIF3B-eGFP) driven by a CMV promoter. Lentiviral particles expressing non-targeting scrambled shRNA (LV-shScrambled-eGFP) were used as a control. To begin, we assessed the in-vivo efficacy of lentiviral particles in knocking down KIF3B in the PrL.

**Fig. 2:**
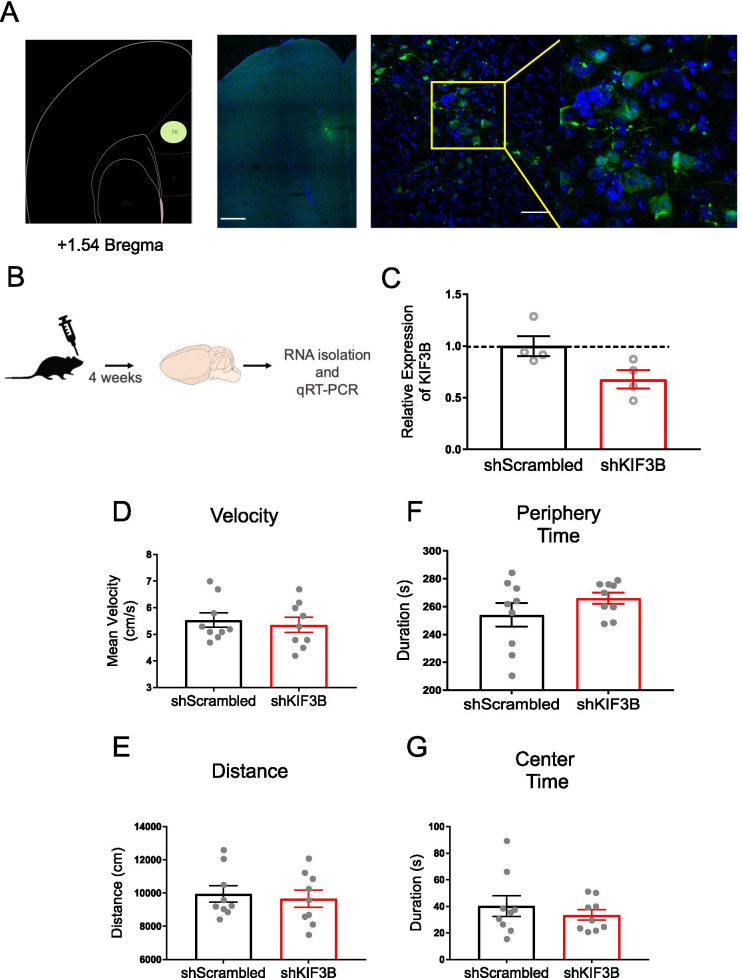
Lentiviral particles expressing KIF3B shRNA in the PrL do not impact locomotor activity or produce anxiety. **A** Representative confocal images of the prelimbic cortices of C56Bl/6 mice injected with 200nL of sh-KIF3B-LV. Virus expressed for four weeks. Enlarged inset (right) shows neuronal-specific labeling. Scale 500 µm, 50 µm. **B** Experimental schema. **C** qRT-PCR analysis of RNA from tissue of the PrL after four weeks of virus expression of shKIF3B. Ct values were normalized to 18 s RNA for dCT and ddCT was calculated relative to the shScambled group levels. Student’s t test. *p < 0.05. Error bars are SEM. N = 4 per group. **D**–**G** Mice were injected with shScrambled-LV and shKIF3B-LV. After four weeks, mice underwent the open field test. Student’s t test. *p < 0.05. Error bars are SEM. N = 9 per group. **F**, **G** Measure of anxiety-like behavior during the first five minutes of the open field test. Data used for preparing plots are shown in Additional file 1: Table S2

Briefly, we injected lentiviral particles of either KIF3B shRNAs or controls into the PrL of nine-week-old mice (Fig. 2A). Four weeks after the injections, the mice were sacrificed, and RNAs were isolated from tissue punches obtained from the PrL (Fig. 2B). As seen in the bar graph, we found that KIF3B expression was significantly reduced with LV-shKIF3B-eGFP injection (shKIF3B-LV 0.68 ± 0.09 and shScrambled-LV 1.00 ± 0.10, Student’s t test, t_6_ = 2.448 *p < 0.05) (Fig. 2C).

We then evaluated whether knockdown of KIF3B within the PrL produced any deleterious effect on the animals by assessing general locomotion and anxiety-like behavior. Four weeks after stereotaxic injection of KIF3B shRNA or control lentiviral particles into the PrL, mice underwent an open field test (OFT) for 30 min. Analysis of the velocity and total distance travelled revealed that shKIF3B-injected mice did not differ from shScrambled-injected mice in general locomotion (velocity: shKIF3B 5.36 cm/s ± 0.28 and shScrambled 5.53 cm/s ± 0.27, Student’s t test, t_16_ = 0.456) (total distance travelled: shKIF3B 9662 cm ± 518.2 and shScrambled 9954 cm ± 489.7, Student’s t test, t_16_ = 0.4101) (Fig. 2D, E). For anxiety-like behavior, shKIF3B mice compared to control mice did not differ in preference for the periphery (shKIF3B 266 s ± 4.048 and shScrambled 254.1 s ± 8.437, Student’s t test, t_16_ = 1.273) or the center (shKIF3B 33.52 s ± 4.019 and shScrambled 40.22 s ± 7.773, Student’s t test, t_16_ = 0.7657) (Fig. 2F, G). This data indicates that knockdown of KIF3B within the PrL does not interfere with locomotion nor does it induce anxiety-like behavior.

### KIF3B knockdown within the PrL impairs long-term memory consolidation

**Fig. 3:**
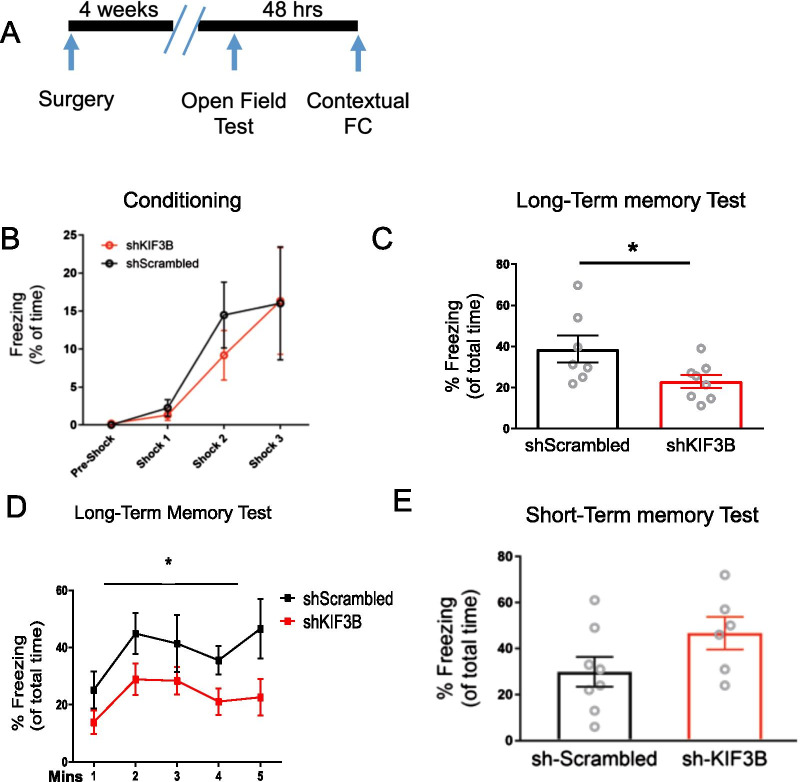
KIF3B knockdown impairs the consolidation of contextual fear memory. **A** Timeline of the behavioral protocol with open field test on day one and contextual fear conditioning on day three. **B** Conditioning data, **C** Long-term memory test, Quantification of the freezing response to the context 24 h after fear conditioning. Student’s t test. *p < 0.05. Error bars are SEM. N = 7 per group. **D** Line graph depicting freezing response to the context 24 h after fear conditioning. Repeated-measures ANOVA; *p < 0.05 with Sidak post-hoc test. Error bars are SEM. N = 7 per group. Data used for preparing plots are shown in Additional file 1: Table S3. **E** KIF3B knockdown has no effect on short-term memory acquisition. Histogram of the freezing response during the short-term memory test performed 1 h. after contextual fear conditioning. Student’s t test. p > 0.05. Error bars are SEM. N = 8 (shScrambled) and 6 (shKIF3B). Data used for preparing plots are shown in Additional file 1: Table S3

We next assessed whether KIF3B knockdown might interfere with the consolidation of contextual fear. To test this, shKIF3B-LV injected mice underwent CFC (Fig. 3A). Mice injected with the LV-shScrambled-eGFP particles were used as a control.

After 24 h of conditioning, fear memory was assessed by measuring the freezing response against the context to which the mice were previously exposed. We found that freezing was significantly impaired in shKIF3B-injected mice as compared to control mice (shKIF3B 22.99% ± 3.228 and shScrambled 38.73% ± 6.559, Student’s t test, t_13_ = 2.243 *p < 0.05; two-way ANOVA with Sidak post-hoc test, F_1,13_ = 5.037 *p < 0.05, Fig. 3B–D), suggesting the important role that KIF3B plays in the consolidation of long-term fear memory.

To determine whether the decreased freezing found in the shKIF3B-injected mice was due to impaired acquisition of fear memory rather that consolidation of fear memory, short term memory acquisition was assessed in shKIF3B-LV and scrambled shRNA mice 1 h after CFC [25]. We found that acquisition was not affected in shKIF3B-injected mice as compared to control mice (shKIF3B 46.67% ± 7.116 and shScrambled 29.88% ± 6.404, Student’s t test, t_13_ = 1.744 p = 0.1068; Fig. 3E) suggesting that KIF3B functions in the consolidation of long-term fear memory rather than its acquisition.

### Knockdown of KIF3B within the PrL impairs freezing during extinction training

**Fig. 4:**
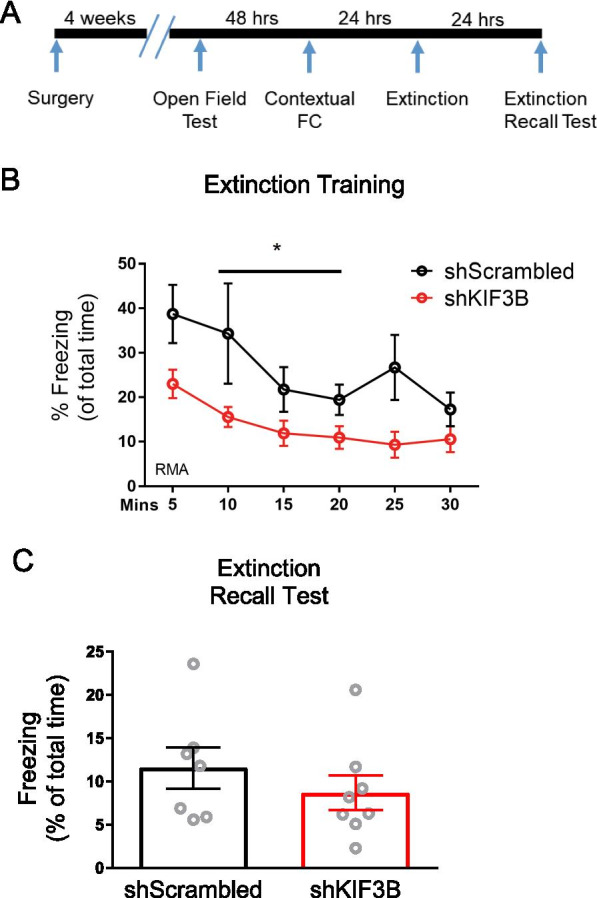
KIF3B knockdown impairs freezing during extinction training. **A** Timeline of the behavioral protocol with extinction training on day four and extinction recall test on day five. **B** Line graph depicting rate of extinction during day four. Repeated-measures ANOVA; *p < 0.05 with Sidak post-hoc test. Error bars are SEM. N = 7 for each group. **C** Histogram of freezing behavior during the extinction recall test. Student’s t test. *p < 0.05. Error bars are SEM. N = 7 per group. Data used for preparing plots are shown in Additional file 1: Table S4

Compelled by recent studies suggesting the PrL’s role in the extinction of contextual fear [7], we next studied how KIF3B depletion in the PrL might impact extinction. Activation of excitatory projections from the PrL to the IL appears to enhance fear extinction [7]. However, the molecular underpinnings of this circuit’s ability to regulate extinction remain unknown. To assess the role of KIF3B expression in the PrL as it pertains to extinction, we carried out mass extinction training of KIF3B shRNA-injected as well as control mice. Mice were then subjected to a 30-min extinction session 24 h after fear conditioning (Fig. 4A). Over six five-minute blocks, the freezing of the KIF3B group was significantly reduced compared to the control group (repeated-measures ANOVA, Sidak post-hoc test, F_1,13_ = 6.284 *p < 0.05) (Fig. 4B). This significance was particularly pronounced in the early and late phases of extinction (i.e., the first and last five minutes of the session).

Next, to determine whether the extinction memory was consolidated, a recall test was performed 24 h after the massed extinction trial. In this recall test, there was no difference in freezing between groups (shKIF3B 8.7% ± 1.97 and shScrambled 11.56% ± 2.40, Student’s t test, t_13_ = 0.93) (Fig. 4C). This therefore indicates that both groups could retain the extinction memory, indicating that KIF3B depletion alters the freezing behavior of mice during extinction training without any effect on extinction recall.

### Knockdown of KIF3B within the PrL increases spine density

**Fig. 5:**
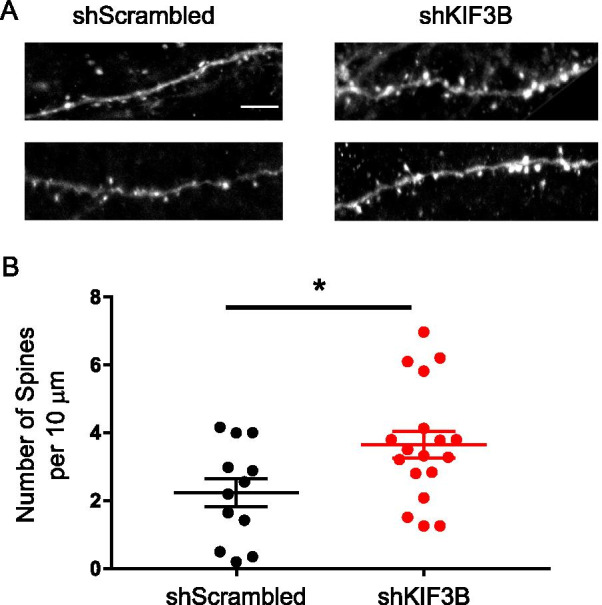
KIF3B knockdown increases the spine density of PrL neurons. **A** Representative confocal image of dendritic segments from the PrL of virus-injected mice after extinction recall. **B** Quantification of spine density. Student’s t test. *p < 0.05. Error bars are SEM. N = 12–18 per group. Scale 5 µm. Data used for preparing plots are shown in Additional file 1: Table S5

To assess whether deficits in memory due to KIF3B depletion can be attributed to a structural change in prelimbic neurons, we next carried out dendritic imaging analysis in the prelimbic cortex. Previous studies have shown that KIF3B plays a role in the structural morphology of neurons [30, 38], and work from our lab has found that KIF3B knockdown in cortical neurons causes an increase in dendritic arborization [30]. We have also found that KIF3B depletion increases the number of mushroom and thin spines [30]. Alsabban et al. found a similar increase in mushroom spines of dissociated hippocampal neurons from KIF3B mutant mice [38]. Cognizant of this work, we hypothesized that KIF3B knockdown would alter the spine density of cortical neurons in vivo, then began testing this concept by performing confocal imaging of dendrites from the PrL. We primarily focused on the layers 2/3 and 5/6 of the prelimbic cortex, as pyramidal neurons in the L2/3 are known to receive strong inputs from the BLA [39], a vital area in fear conditioning and fear extinction. Quantitative analysis of the images showed that KIF3B knockdown significantly increased the spine density of PrL neurons (shKIF3B 3.647 ± 0.40 and shScrambled 2.241 ± 0.42, Student’s t test, t_28_ = 2.36 *p < 0.05) (Fig. 5A, B), validating our hypothesis.

## Discussion

Molecular and cellular mechanisms underlying memory consolidation remain elusive. Despite our understanding of the key role of PrL in contextual fear memory consolidation [25], the molecular mechanisms underlying the PrL’s function remain unclear. Here, we have investigated the role of KIFs in prelimbic neurons during the consolidation of contextual fear. It has been shown that KIFs are physiologically regulated in sensory and motor neurons of gill withdrawal reflex of sea slug Aplysia [32, 40] and that expression of KIF5C in CA1 neurons of dorsal hippocampus hinder the consolidation of contextual fear memory [41]. However, the role of KIFs in PrL neurons in mediating memory consolidation is not known.

We first examined whether CFC could modulate the expression of KIFs. Consistent with our earlier findings on the regulation of specific KIFs in Aplysia [32] and mouse hippocampus [41], we found that among the 12 KIFs studied, KIF3B and KIF5A were upregulated in the prelimbic cortex after CFC, but not in the infralimbic cortex (Fig. 1). Taken together, these results suggest neuronal-specific regulation of KIFs during learning. Guided by our earlier work identifying KIF3B as a key regulator of neuronal morphology [30], we focused on KIF3B and carried out loss-of-function studies in the PrL. We have previously shown that KIF3B depletion in cortical neurons enhances dendritic architecture, a similar finding to that observed with KIF11 depletion [29], and that KIF3B depletion also causes an increase in mushroom and thin spines [30].

Consistent with our assumption that KIFB upregulation could prove critical for the consolidation of contextual fear, KIF3B depletion in the PrL impaired the consolidation of contextual fear memory, while leaving locomotion and the acquisition of fear memory intact (Fig. 3). KIF3B depletion also did not induce anxiety-like behavior (Fig. 3). Furthermore, KIF3B depletion appears to reduce freezing during extinction training, but not during extinction recall (Fig. 4). These results suggest that KIF3B promotes the consolidation of contextual fear memory, while it may also inhibit extinction learning.

It is important to note that given the degree of impairment in fear consolidation, the impaired freezing during extinction learning could be due to a weakening of the fear memory. This possibility is noteworthy considering that extinction recall was not affected. Additional studies in which KIF3B is depleted immediately before extinction training would be required to determine the exact impact of KIF3B on extinction. Since the behavior training was conducted 4 weeks after lentivirus injections, manipulation of KIF3B just prior to extinction training was not possible in this study.

KIF3B could be mediating the observed behavioral effects by impeding spine density. It is important to recall that the PrL receives robust projections from the basolateral amygdala [39], as well as from the dorsal [42] and ventral [43] hippocampus. The hippocampus relays critical contextual information to the prelimbic cortex. Therefore, the impaired freezing observed in our studies could be due to a disruption of these circuits, as evidenced by the altered spine density in PrL neurons with KIF3B knockdown. It remains to be determined how structural changes in KIF3B-deficient neurons impact PrL and IL projections that modulate fear expression and fear extinction, respectively. Furthermore, due to technical limitations, we were unable to determine the morphology of the spines in PrL neurons after KIF3B depletion. This would be interesting to study in future work as previous studies have found that KIF3B knockdown increases the number of mushroom and thin spines [30, 38].

Our spine findings are in line with previous research. It has been demonstrated that fear conditioning and extinction have opposing effects on dendritic spine remodeling [44, 45], with Lai et al. showing that fear conditioning induces dendritic spine elimination whereas extinction increases spine formation. This raises the possibility that the observed impairment in fear conditioning could be due to a failure to eliminate spines under KIF3B knockdown.

There are several neurological disorders in which extinction failure is a prominent feature. These disorders include PTSD, which is characterized by intrusive and aversive thoughts that are resistant to extinction [46]. The current prevalence of disorders like PTSD provides a strong clinical relevance for understanding the neural underpinnings of fear memory formation and extinction. The results presented here raise the possibility that KIF3B depletion could be leveraged therapeutically to either impair traumatic fear memories or promote their extinction. This has the potential to lead to the ultimate abolishment of traumatic fear memories. One logical question that subsequently arises here is whether KIF3B knockdown can block traumatic memories and enhance extinction in animal models of PTSD.

## Conclusions

In summary, our data represent a significant initial step to delineate the mechanisms within the PrL involving the expression and extinction of contextual memories. These data provide confirmation for the transcriptional regulation of KIFs in the PrL and offers early proof implicating KIFs in extinction. This work also contributes to the growing body of evidence suggesting that the PrL is actively involved in extinction [7]. Finally, this work is the first to show that KIF3B is involved in the regulation of contextual fear memory, opening an avenue for additional studies on KIFs aimed at solving the major clinical and societal burden posed by PTSD and similar conditions.

## Materials and methods

### Animals

Nine to 12-week-old male C57BL6/J mice (Jackson Laboratories, Bar Harbor, ME) were housed in groups of four or five on a 12-h light/dark cycle with ad libitum access to food and water. All experiments were performed during the light phase of the diurnal cycle.

### Laser capture dissection

The laser capture microdissection was carried out as described in Kadakkuzha et al. [47]. Nine-week-old C57BL6/J mice underwent contextual fear conditioning. One hour later, the mice were sacrificed, and the brains were freshly frozen in OCT compound. The brains were then sectioned at 14 µm on a Leica 3050 s cryostat, saving every other section throughout the prefrontal cortex. Sections were immediately mounted unto PEN membrane slides. Next, the sections were stained with Cresyl Violet using the LCM staining kit (Cat# AM1935, Life Technologies) according to the manufacturer’s instructions. Following staining, the slides were stored at room temperature until the laser capture microdissection (LCM) procedure. Microdissection of the PrL and IL were performed using the Leica LMD7000 LCM microscope, in which laser power was set at 50 mW and each capture was performed with two or fewer laser pulses. After the microdissection, each sample was placed in 50 µl of ice-cold lysis buffer. The RNA was extracted using the Arcturus™ PicoPure™ RNA Isolation Kit (Cat#1220-01, Applied Biosystems) according to the manufacturer’s instructions.

### Quantitative PCR (qPCR)

qPCR was carried out as described previously [48]. Quantification of each transcript was normalized to the mouse 18S reference gene following the 2^−ΔΔCt^ method [49]. Student’s t test or one-way ANOVA was used to analyze genes with statistically significant expression levels, where *p-value < 0.05.

### Production of lentiviral particles for in-vivo analysis

LV-shKIF3B-eGFP and LV-shScrambled-eGFP were packaged and concentrated as previously described [30] with the following exceptions. HEK-293T cells were transfected with TransIT-VirusGEN transfection reagent (Cat #MIR 6703**,** Mirus). After recovery, the viruses were titrated using the protocol from Tiscornia et al. [50]. High titers (> 10^9^ TU/ml) were acquired for both LV-shKIF3B-eGFP and LV-shScrambled-eGFP.

### Surgical procedure

For behavior experiments, mice were anesthetized with isoflurane (induction 3%; surgery 1.5–1.8%). Animals were then placed in a stereotaxic frame. The scalp was shaved, and the skin was disinfected with sequential swabs of surgical scrub (Nolvasan Surgical Scrub) and Betadine. A small incision was made to the midline of the head. Small holes were drilled into the skull using a robotic surgery apparatus (Cambridge NeuroTech). Then, mice were bilaterally injected with 200 nl of LV-shKIF3B-eGFP using the following coordinates PrL: A/P + 2.3, M/L ± 0.2, D/V 2.5. The incision was closed with Vetbond Tissue Adhesive and the mice were injected subcutaneously with 1 mg/kg of Baytril and Metacam diluted in saline. The mice were then returned to their home cage, where they remained for four weeks to allow the virus to express. Mice with unilateral, off target, or no viral expression were excluded from analysis.

### Behavioral procedures

#### Open field test

Four weeks after surgery, mice were brought to a dimly lit room with white noise set to 70 dB to mask any noises. The mice were placed in acrylic chambers with white backgrounds placed on the walls to prevent them being viewed by other mice. Movement was tracked from overhead using an infrared CCD camera paired to a computer running EthoVision 15 software (EthoVision 15; Noldus Information Technology, Leesburg, VA; http://www.noldus.com/ethovision). Each trial lasted 30 min and then the mice were immediately returned to their home cage.

#### Contextual fear conditioning

Contextual fear conditioning experiments were performed using a set of four modified Noldus PhenoTyper (Model 3000) chambers (Leesburg, VA) with shock floors [25]. Automated tracking and shock delivery were performed using EthoVision 15 software (EthoVision 15; Noldus Information Technology, Leesburg, VA; http://www.noldus.com/ethovision). Each chamber was cleaned with 70% ethanol prior to each trial. The chambers were illuminated with white light throughout training and testing and 72 dB white noise was played in the room to mask any unintended noise that might add to the context. During the training session, mice received three two-second 0.50 mA scrambled foot shocks, 2.5, 3.5, and 4.5 min after placement into the chamber. Mice were promptly removed from the chamber after 5.5 min. Shock-only control mice were given a 2 s foot shock immediately after being placed in the chamber and were quickly returned to the home cage [25]. Freezing behavior was measured as the percentage of time spent freezing (defined as immobility except for respiration) [51]. To test effect of KIF3B on fear memory acquisition, KIF3B shRNA or nontargeting control shRNA-injected mice were returned to the conditioning chambers, one hour after training. Freezing behavior was assessed for 5 min in the absence of shock.

#### Extinction

Twenty-four hours after training, the mice were returned to the chambers for massed extinction training [10]. Mice remained in the chambers for 30 min without shock. After the trial, the mice were immediately returned to their home cage. To test whether the mice retained the extinction learning, an extinction recall test was performed 24 h later. The mice were returned to the same chamber without shock for a 5-min trial.

### Immunohistochemistry

Four weeks after surgery, mice were anesthetized with sodium pentobarbital (Fatal Plus, 40 mg/kg) and transcardially perfused with PBS followed by freshly prepared 4% PFA in PBS. The brains were harvested and post-fixed in the same fixative at 4 °C overnight. The brains were transferred to a 30% sucrose solution at 4 °C until fully saturated. The brains were frozen in OCT compound, sectioned (25 µm) on a cryostat, and mounted on slides. The slices were fixed with 4% PFA in PBS for 10 min. Then, the slices were permeabilized with 0.3% Triton X-100 in PBS for 8 min at room temperature. The slices were then washed 3 times with PBS and blocked with 10% normal horse serum (NHS) in PBS for 1 h at room temperature. Then, the slices were incubated with GFP antibody (1:3000, NB100-1614, Novus Biologicals) at 4 °C for 16–18 h in 5% NHS and 0.3% Triton X-100 in PBS. Sections were washed five times for 12 min each with 0.3% Triton X-100 in PBS. The sections were then incubated with Alexa-488 (1:2000, Jackson ImmunoResearch, Inc.) for 1 h at room temperature in the dark. Slices were washed five times for 12 min in 0.3% Triton X-100 in PBS. The slices were rinsed with PBS and mounted with Fluoro-Gel with DAPI (EMS).

### Spine imaging and reconstruction

Following immunohistochemistry, high-resolution images of the prelimbic cortical neuronal branches were acquired with a Zeiss 780 confocal microscope. Images were taken with a 63X water immersion lens (N.A. 1.3). 1.0X scan zoom was used to take a *z*-stack through the depth of the section. Imaged dendrites were at a low enough density to provide accurate resolution of individual dendritic spines. Approximately 20–30 z-stack images were taken per section, with a *z*-distance between two serial images of 0.25 μm. The images were then imported into the Zen software for analysis. To quantify spine density, 1–3 dendrites, ranging from 15 to 50 μm in length and less than 1 μm in width, were chosen for analysis (7 animals per group). Dendritic segments with a width over 1 μm are classified as primary branches, while those under 1 μm are secondary and tertiary according to size. Since previous research has found that secondary and tertiary branches are more receptive to perturbation (i.e., learning and stress), they were chosen for analysis [52]. Dendritic spines were distinguished from filopodia by choosing dendritic protrusions with the following criteria: ratio of head diameter to neck diameter > 1.2:1 and ratio of length to neck diameter < 3:1, as described in Young et al. [52]. Due the resolution of the images, we were unable to convincingly determine the morphology of the spines.

### Statistics

All statistical analysis was performed with Prism 7 software (GraphPad)**.** Student’s *t* test, one-way ANOVA, or repeated-measures ANOVA was used for comparison between the groups, and statistical significance was defined as p < 0.05. Outliers were identified with a Grubbs test Q = 5%.

## Supplementary Information

**Additional file 1.** Data and statistical analyses used for preparing the figures in the manuscript.

## Data Availability

Data and materials described in the current study are available from the corresponding author on reasonable request.

## References

[1] LeDoux JE (2000). Emotion circuits in the brain. Annu Rev Neurosci.

[2] Rozeske RR, Valerio S, Chaudun F, Herry C (2015). Prefrontal neuronal circuits of contextual fear conditioning. Genes Brain Behav.

[3] Kim JJ, Jung MW (2006). Neural circuits and mechanisms involved in Pavlovian fear conditioning: a critical review. Neurosci Biobehav Rev.

[4] Bouton ME, Westbrook RF, Corcoran KA, Maren S (2006). Contextual and temporal modulation of extinction: behavioral and biological mechanisms. Biol Psychiatry.

[5] Bouton ME (2004). Context and behavioral processes in extinction. Learn Memory.

[6] Kim HS, Cho HY, Augustine GJ, Han JH (2016). Selective control of fear expression by optogenetic manipulation of infralimbic cortex after extinction. Neuropsychopharmacology.

[7] Marek R, Xu L, Sullivan RKP, Sah P (2018). Excitatory connections between the prelimbic and infralimbic medial prefrontal cortex show a role for the prelimbic cortex in fear extinction. Nat Neurosci.

[8] Kim SC, Jo YS, Kim IH, Kim H, Choi J-S (2010). Lack of medial prefrontal cortex activation underlies the immediate extinction deficit. J Neurosci.

[9] Sun W, Li X, An L (2018). Distinct roles of prelimbic and infralimbic proBDNF in extinction of conditioned fear. Neuropharmacology.

[10] Mamiya N, Fukushima H, Suzuki A, Matsuyama Z, Homma S, Frankland PW, Kida S (2009). Brain region-specific gene expression activation required for reconsolidation and extinction of contextual fear memory. J Neurosci.

[11] Knapska E, Macias M, Mikosz M, Nowak A, Owczarek D, Wawrzyniak M, Pieprzyk M, Cymerman IA, Werka T, Sheng M, Maren S, Jaworski J, Kaczmarek L (2012). Functional anatomy of neural circuits regulating fear and extinction. Proc Natl Acad Sci U S A.

[12] Milad MR, Quirk GJ (2002). Neurons in medial prefrontal cortex signal memory for fear extinction. Nature.

[13] Herry C, Mons N (2004). Resistance to extinction is associated with impaired immediate early gene induction in medial prefrontal cortex and amygdala. Eur J Neurosci.

[14] Giustino TF, Maren S (2015). The role of the medial prefrontal cortex in the conditioning and extinction of fear. Front Behav Neurosci.

[15] Quirk GJ, Garcia R, Gonzalez-Lima F (2006). Prefrontal mechanisms in extinction of conditioned fear. Biol Psychiatry.

[16] Baldi E, Bucherelli C (2015). Brain sites involved in fear memory reconsolidation and extinction of rodents. Neurosci Biobehav Rev.

[17] Burgos-Robles A, Vidal-Gonzalez I, Quirk GJ (2009). Sustained conditioned responses in prelimbic prefrontal neurons are correlated with fear expression and extinction failure. J Neurosci.

[18] Vidal-Gonzalez I, Vidal-Gonzalez B, Rauch SL, Quirk GJ (2006). Microstimulation reveals opposing influences of prelimbic and infralimbic cortex on the expression of conditioned fear. Learn Mem.

[19] Sierra-Mercado D, Padilla-Coreano N, Quirk GJ (2011). Dissociable roles of prelimbic and infralimbic cortices, ventral hippocampus, and basolateral amygdala in the expression and extinction of conditioned fear. Neuropsychopharmacology.

[20] Corcoran KA, Quirk GJ (2007). Activity in prelimbic cortex is necessary for the expression of learned, but not innate, fears. J Neurosci.

[21] Laurent V, Westbrook RF (2009). Inactivation of the infralimbic but not the prelimbic cortex impairs consolidation and retrieval of fear extinction. Learn Memory.

[22] Choi DC, Maguschak KA, Ye K, Jang SW, Myers KM, Ressler KJ (2010). Prelimbic cortical BDNF is required for memory of learned fear but not extinction or innate fear. Proc Natl Acad Sci U S A.

[23] Siddiqui SA, Singh S, Ranjan V, Ugale R, Saha S, Prakash A (2017). Enhanced histone acetylation in the infralimbic prefrontal cortex is associated with fear extinction. Cell Mol Neurobiol.

[24] Santini E, Quirk GJ, Porter JT (2008). Fear conditioning and extinction differentially modify the intrinsic excitability of infralimbic neurons. J Neurosci.

[25] Rizzo V, Touzani K, Raveendra BL, Swarnkar S, Lora J, Kadakkuzha BM, Liu XA, Zhang C, Betel D, Stackman RW, Puthanveettil SV (2017). Encoding of contextual fear memory requires de novo proteins in the prelimbic cortex. Biol Psychiatry Cogn Neurosci Neuroimaging..

[26] Lamprecht R, LeDoux J (2004). Structural plasticity and memory. Nat Rev Neurosci.

[27] Bailey CH, Kandel ER, Harris KM (2015). Structural components of synaptic plasticity and memory consolidation. Cold Spring Harb Perspect Biol.

[28] Cole CJ, Mercaldo V, Restivo L, Yiu AP, Sekeres MJ, Han JH, Vetere G, Pekar T, Ross PJ, Neve RL, Frankland PW, Josselyn SA (2012). MEF2 negatively regulates learning-induced structural plasticity and memory formation. Nat Neurosci.

[29] Swarnkar S, Avchalumov Y, Raveendra BL, Grinman E, Puthanveettil SV (2018). Kinesin family of proteins Kif11 and Kif21B act as inhibitory constraints of excitatory synaptic transmission through distinct mechanisms. Sci Rep.

[30] Joseph NF, Grinman E, Swarnkar S, Puthanveettil SV (2020). Molecular motor KIF3B acts as a key regulator of dendritic architecture in cortical neurons. Front Cell Neurosci.

[31] Muhia M, Thies E, Labonte D, Ghiretti AE, Gromova KV, Xompero F, Lappe-Siefke C, Hermans-Borgmeyer I, Kuhl D, Schweizer M, Ohana O, Schwarz JR, Holzbaur ELF, Kneussel M (2016). The kinesin KIF21B regulates microtubule dynamics and is essential for neuronal morphology, synapse function, and learning and memory. Cell Rep.

[32] Puthanveettil SV, Monje FJ, Miniaci MC, Choi Y-B, Karl KA, Khandros E, Gawinowicz MA, Sheetz MP, Kandel ER (2008). A new component in synaptic plasticity: upregulation of kinesin in the neurons of the gill-withdrawal reflex. Cell.

[33] Yin X, Takei Y, Kido MA, Hirokawa N (2011). Molecular motor KIF17 is fundamental for memory and learning via differential support of synaptic NR2A/2B levels. Neuron.

[34] Hirokawa N, Noda Y, Tanaka Y, Niwa S (2009). Kinesin superfamily motor proteins and intracellular transport. Nat Rev Mol Cell Biol.

[35] Freixo F, Martinez Delgado P, Manso Y, Sanchez-Huertas C, Lacasa C, Soriano E, Roig J, Luders J (2018). NEK7 regulates dendrite morphogenesis in neurons via Eg5-dependent microtubule stabilization. Nat Commun.

[36] van der Vaart B, van Riel WE, Doodhi H, Kevenaar Josta T, Katrukha Eugene A, Gumy L, Bouchet Benjamin P, Grigoriev I, Spangler Samantha A, Yu Ka L, Wulf Phebe S, Wu J, Lansbergen G, van Battum EY, Pasterkamp RJ, Mimori-Kiyosue Y, Demmers J, Olieric N, Maly Ivan V, Hoogenraad Casper C, Akhmanova A (2013). CFEOM1-associated kinesin KIF21A is a cortical microtubule growth inhibitor. Dev Cell.

[37] van Riel WE, Rai A, Bianchi S, Katrukha EA, Liu Q, Heck AJR, Hoogenraad CC, Steinmetz MO, Kapitein LC, Akhmanova A (2017). Kinesin-4 KIF21B is a potent microtubule pausing factor. Elife.

[38] Alsabban AH, Morikawa M, Tanaka Y, Takei Y, Hirokawa N (2019). Kinesin Kif3b mutation reduces NMDAR subunit NR2A trafficking and causes schizophrenia-like phenotypes in mice. EMBO J.

[39] Cheriyan J, Kaushik MK, Ferreira AN, Sheets PL (2016). Specific targeting of the basolateral amygdala to projectionally defined pyramidal neurons in prelimbic and infralimbic cortex. J eNeuro..

[40] Puthanveettil S, Kandel E, Curran T, Christen Y (2011). Molecular mechanisms for the initiation and maintenance of long-term memory storage. Two faces of evil cancer and neurodegeneration.

[41] Swarnkar S, Avchalumov Y, Espadas I, Grinman E, Liu XA, Raveendra BL, Zucca A, Mediouni S, Sadhu A, Valente S, Page D, Miller K, Puthanveettil SV (2021). Molecular motor protein KIF5C mediates structural plasticity and long-term memory by constraining local translation. Cell Rep.

[42] Ye X, Kapeller-Libermann D, Travaglia A, Inda MC, Alberini CM (2017). Direct dorsal hippocampal-prelimbic cortex connections strengthen fear memories. Nat Neurosci.

[43] Meyer HC, Odriozola P, Cohodes EM, Mandell JD, Li A, Yang R, Hall BS, Haberman JT, Zacharek SJ, Liston C, Lee FS, Gee DG (2019). Ventral hippocampus interacts with prelimbic cortex during inhibition of threat response via learned safety in both mice and humans. Proc Natl Acad Sci.

[44] Lai CSW, Adler A, Gan W-B (2018). Fear extinction reverses dendritic spine formation induced by fear conditioning in the mouse auditory cortex. Proc Natl Acad Sci.

[45] Lai CSW, Franke TF, Gan W-B (2012). Opposite effects of fear conditioning and extinction on dendritic spine remodelling. Nature.

[46] Loayza Careaga MB, Neves Girardi CE, Suchecki D (2016). Understanding posttraumatic stress disorder through fear conditioning, extinction and reconsolidation. Neurosci Biobehav Rev.

[47] Kadakkuzha BM, Liu XA, McCrate J, Shankar G, Rizzo V, Afinogenova A, Young B, Fallahi M, Carvalloza AC, Raveendra B, Puthanveettil SV (2015). Transcriptome analyses of adult mouse brain reveal enrichment of lncRNAs in specific brain regions and neuronal populations. Front Cell Neurosci.

[48] Graff J, Joseph NF, Horn ME, Samiei A, Meng J, Seo J, Rei D, Bero AW, Phan TX, Wagner F, Holson E, Xu J, Sun J, Neve RL, Mach RH, Haggarty SJ, Tsai LH (2014). Epigenetic priming of memory updating during reconsolidation to attenuate remote fear memories. Cell.

[49] Livak KJ, Schmittgen TD (2001). Analysis of relative gene expression data using real-time quantitative PCR and the 2(-Delta Delta C(T)) Method. Methods.

[50] Tiscornia G, Singer O, Verma IM (2006). Production and purification of lentiviral vectors. Nat Protoc.

[51] Blanchard RJ, Blanchard DC (1969). Crouching as an index of fear. J Comp Physiol Psychol.

[52] Young EJ, Aceti M, Griggs EM, Fuchs RA, Zigmond Z, Rumbaugh G, Miller CA (2014). Selective, retrieval-independent disruption of methamphetamine-associated memory by actin depolymerization. Biol Psychiatry.

